# Association between cyclin D1 (CCND1) G870A polymorphism and gastric cancer risk: a meta-analysis

**DOI:** 10.18632/oncotarget.11848

**Published:** 2016-09-06

**Authors:** Yafei Zhang, Xianling Zeng, Hongwei Lu, Hong Ji, Enfa Zhao, Yiming Li

**Affiliations:** ^1^ Department of General Surgery, Second Affiliated Hospital, School of Medicine, Xi'an Jiaotong University, Xi'an, Shaanxi, China; ^2^ Department of Obstetrics and Gynecology, First Affiliated Hospital, School of Medicine, Xi'an Jiaotong University, Xi'an, Shaanxi, China; ^3^ Department of Ultrasound, Second Affiliated Hospital, School of Medicine, Xi'an Jiaotong University, Xi'an, Shaanxi, China

**Keywords:** gastric cancer, cyclin D1 (CCND1) G870A, polymorphism, meta-analysis

## Abstract

Published data on the association between cyclin D1 (CCND1) G870A polymorphism and gastric cancer (GC) risk are inconclusive. Thus, we conducted a meta-analysis to evaluate the relationship between CCND1 G870A polymorphism and GC risk. We searched PubMed, EMBASE, Web of science and the Cochrane Library up to June 12, 2015 for relevant studies. Odds ratios (ORs) and 95% confidence intervals (CIs) were used to estimate the strength of associations. Nine studies published from 2003 to 2014, with a total of 1813 cases and 2173 controls, were included in this meta-analysis. The pooled results showed that there was no association between CCND1 G870A polymorphism and GC risk in any genetic model. The subgroup analysis stratified by ethnicity showed an increased breast cancer risk in Caucasian based on heterozygote comparison (GA vs. GG: OR=1.49, 95% CI=1.06-2.10, P=0.02). We found the same association in population based (PB) stratified analyses by Source of controls (AA vs. GG: OR=1.39, 95% CI=1.01-1.93, 0.05). When stratifying by the type, Sex and H. pylori infection in dominant model, Interestingly, we found the opposite result in Male (AA + GA vs. GG: OR=0.5, 95% CI=0.33-0.76, P=0.001), there were no association between CCND1 G870A polymorphism and GC risk in any other subgroup. This meta-analysis suggests that CCND1 G870A polymorphism is a risk factor for susceptibility to GC in Caucasians and in general populations. While, CCND1 G870A polymorphism plays a possible protective effect in GC in Male. Further large scale multicenter epidemiological studies are warranted to confirm this finding.

## INTRODUCTION

Gastric cancer(GC), one of the most frequently encountered malignant tumors, has become the third main reasons of tumor-associated death in our word, whose 5-year survival rate is low, especially for advanced GC [1, 2]. In most of non developed world, the incidence of GC is constantly increasing, as well as mortality [3, 4]. For most GCs are diagnosed to be advanced stages, early detection seems particularly important [5]. While, the determination of the relationship between CCND1 G870A polymorphism and the occurrence of GC provides us an effective way to reach the goal.

As a kind of important proteins that regulate cell cycle, CCND1 is of important effect in the regulation of cell transformation from G1 phase to S phase [6, 7]. In exon 4, CCND1 gene has a G > A polymorphism (G870A), which makes mRNA to produce an alternative splice site, change the protein structure of the carboxy terminal domain, resulting the disorder in cell cycle regulation Checkpoint (G1/S), reduced the capacity of DNA repair [8, 9]. Over expression of related proteins will accelerate the G1 phase, and promote the proliferation of cells, which may lead to cancer occurrence [10, 11].

Previous functional studies have reported the relationship between cyclin D1 G870A polymorphism and the occurrence of GC, However, the conclusions are still inconclusive [12–20]. To clarify this, Chen et al [21] made a comprehensive analysis of the associations between cyclin D1 G870A and digestive tract cancers. However, number of their studies included in their meta-analysis about GC is just four, and GC is just a small part of their study. In their subgroup studies, the sample size is extremely small. Therefore, we decided to carry out a meta-analysis on the whole included case-control researches to make a more accurate assessment of the relationship. Furthermore, we conducted several subgroup analyses stratified by ethnicity, source of controls, genotyping method, tumor type, Sex and H. pylori infection.

## RESULTS

### Characteristics of eligible studies

**Figure 1:**
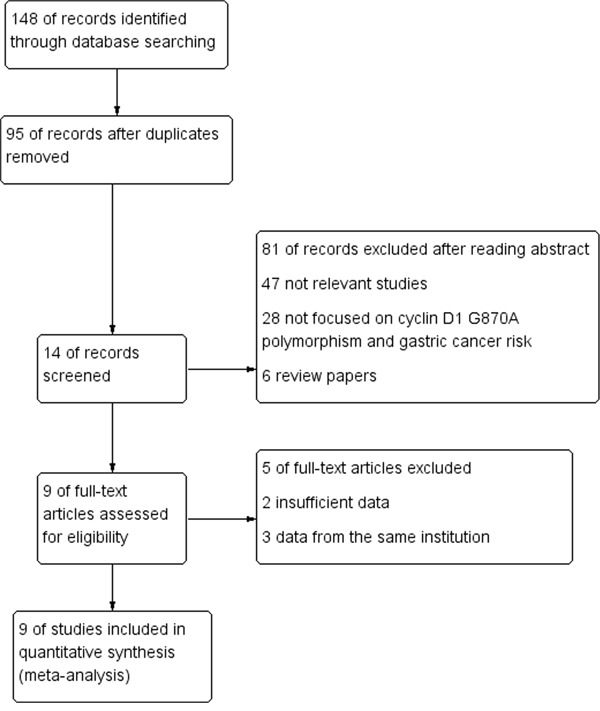
Flow chart of studies selection in this meta-analysis

Detailed retrieval procedures are summarized in Figure 1. A total of 148 references were preliminarily identified at first based on our selection strategy. There were 28, 51, 68, 1 records in database of PubMed, EMBASE, Web of science and the Cochrane Library, respectively. 95 records left after excluding duplicate articles. We reviewed titles and abstracts of all identified studies and excluded 47 papers that were clearly irrelevant, 28 studies that not focused on CCND1 G870A polymorphism and the occurrence of GC, 6 records that were review papers. Next, the whole of the rest of the papers were examined according to the inclusion and exclusion criteria. 5 of full-text articles excluded for 2 insufficient data and 3 data from the same institution. Finally, 9 studies about cyclin D1 G870A polymorphism and GC risk were eventually included in our study, including 1813 cases and 2173 controls. Characteristics of eligible analyses are shown in Table 1. The 9 case–control papers were published between 2003 and 2014, among them, 2 studies were performed in Caucasians and 7 in Asians. Four studies were hospital-based, four were population-based and one not reported.

**Table 1:** Characteristics of the studies included in the meta-analysis

First author	Year	Country	Ethnicity	Study design	Source of controls	Genotyping method	Number(case/control)	HWE
Zhang et al [12]	2003	China	Asian	CC	PB	PCR-SSCP	87/183	0.117904
Kiel et al [16]	2004	Germany	Caucasian	CC	PB	PCR-RFLP	106/245	0.216215
Geddert et al [18]	2005	Germany	Caucasian	CC	HB	PCR-RFLP	286/253	0.223914
Song et al [14]	2007	Korea	Asian	CC	NR	PCR-SSCP	253/442	0.623066
Jia et al [17]	2008	China	Asian	CC	HB	PCR-RFLP	159/162	0.080933
Fang et al [19]	2013	China	Asian	CC	HB	PCR-RFLP	115/112	0.2067
Tahara et al [13]	2009	Japan	Asian	CC	HB	PCR-RFLP	392/359	0.923934
Bukum et al [20]	2013	Turkey	Asian	CC	PB	PCR-RFLP	57/59	0.634847
Kuo et al [15]	2014	China	Asian	CC	PB	PCR-RFLP	358/358	0.000288

HWE: Hardy-Weinberg equilibrium; CC: case-control; PB: population based; HB: hospital-based; NR: not reported; PCR: polymerase chain reaction; RFLP: restriction fragment length polymorphism; SSCP: Single Strand Conformation Polymorphism;

### Meta-analysis results

**Figure 2:**
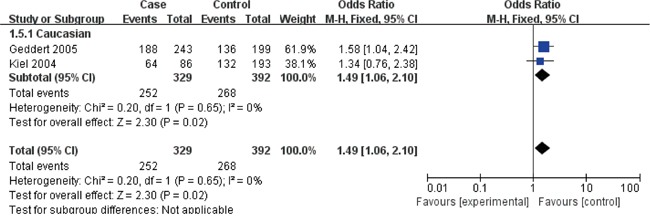
Forest plots of the cyclin D1 (CCND1) G870A polymorphism and gastric cancer risk in the Caucasian subgroup (GA vs. GG) Notes: The squares and horizontal lines correspond to the study specific OR and 95% CI. The area of the squares reflects the weight (inverse of the variance). The diamond represents the summary OR and 95% CI.Abbreviations: CI, confidence interval; OR, odds ratio; df, degrees of freedom; M-h, Mantel-haenszel.

**Figure 3:**
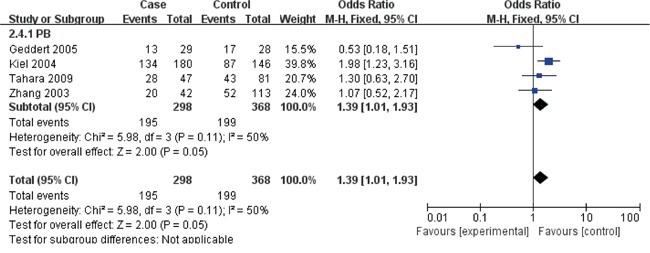
Forest plots of the cyclin D1 (CCND1) G870A polymorphism and gastric cancer risk in the population based (PB) subgroup (AA vs. GG) Abbreviations: CI, confidence interval; OR, odds ratio; df, degrees of freedom; M-h, Mantel-haenszel.

**Figure 4:**
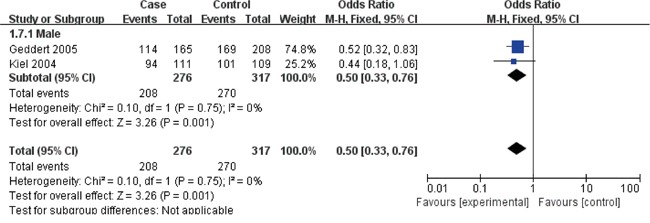
Forest plots of the cyclin D1 (CCND1) G870A polymorphism and gastric cancer risk in the Male subgroup (AA + GA vs. GG) Abbreviations: CI, confidence interval; OR, odds ratio; df, degrees of freedom; M-h, Mantel-haenszel.

The Cyclin D1 (G870A) polymorphisms genotype distribution and allele frequency in cases and controls were listed in Table 2. The main results of our study were shown in Table 3 and 4. A total of 9 studies with 1813 cases and 2173 controls were included. As show in Table 3, The pooled results indicated that there was not any relationships between G870A polymorphism and the occurrence of GC in any genetic model: Allele model (OR=1.07, 95% CI=0.88-1.30, P=0.51), dominant model (OR=1.07, 95% CI=0.81-1.41, P=0.65) recessive model (OR=1.09, 95% CI=0.80-1.49, P=0.58) homozygous genetic model (OR=1.09, 95% CI=0.73-1.63, P=0.66) heterozygote comparison (OR=1.03, 95% CI=0.80-1.32, P=0.81). The subgroup analysis stratified by ethnicity showed an increased GC risk in Caucasian based on heterozygote comparison (Figure 2, OR=1.49, 95% CI=1.06-2.10, P=0.02). while, there was not any genetic models attained statistical correlation in Asians (Table 3). We found an increased GC risk in population based (PB) stratified analyses by Source of controls (Figure 3, homozygous genetic model: OR=1.39, 95% CI=1.01-1.93, 0.05). However, no statistically significant association in hospital-based (HB) (Table 3). When stratifying by the type, Sex and H. pylori infection in dominant model, Interestingly, we found the opposite result in Male (Figure 4, dominant model: OR=0.5, 95% CI=0.33-0.76, P=0.001). While, not any relationships between CCND1 G870A polymorphism and GC risk in any other subgroups (Table 4).

**Table 2:** Cyclin D1 (G870A) polymorphisms genotype distribution and allele frequency in cases and controls

First author	Genotype (N)								Allele frequency (N)			
	Case				Control				Case		Control	
	Total	GG	GA	AA	Total	GG	GA	AA	G	A	G	A
Zhang et al [12]	87	19	40	28	183	38	102	43	78	96	178	188
Kiel et al [16]	106	22	64	20	245	61	132	52	108	104	254	236
Geddert et al [18]	286	55	188	43	253	63	136	54	298	274	262	244
Song et al [14]	253	71	125	57	442	102	226	114	267	239	430	454
Jia et al [17]	159	31	81	47	162	16	85	61	143	175	117	207
Fang et al [19]	115	17	46	52	112	36	49	27	80	150	121	103
Tahara et al [13]	392	98	197	97	359	98	180	81	393	391	376	342
Bukum et al [20]	57	16	28	13	59	11	31	17	60	54	53	65
Kuo et al [15]	358	46	178	134	358	59	212	87	270	446	330	386

**Table 3:** Meta-analysis results

subgroup		OR	95%CI	P value	Heterogeneity		Effects model
					I^2^	P value	
A vs. G							
Overall		1.07	0.88-1.30	0.51	77%	<0.0001	R
Ethnicity	Caucasian	1	0.83-1.22	0.96	0%	0.81	F
	Asian	1.09	0.84-1.41	0.53	83%	<0.0001	R
Source of controls	PB	1.13	0.88-1.44	0.34	54%	0.09	R
	HB	1.12	0.77-1.61	0.56	86%	<0.0001	R
Genotyping method	PCR-SSCP	0.92	0.77-1.11	0.40	54%	0.14	F
	PCR-RFLP	1.10	0.86	1.40	80%	<0.0001	R
AA + GA vs. GG							
Overall		1.07	0.81-1.41	0.65	66%	0.003	R
Ethnicity	Caucasian	1.35	0.97-1.87	0.08	0%	0.79	F
	Asian	0.99	0.70-1.41	0.96	71%	0.002	R
Source of controls	PB	1.13	0.86-1.49	0.39	9%	0.35	F
	HB	1.19	0.69-2.05	0.54	81%	0.001	R
Genotyping method	PCR-SSCP	0.81	0.59-1.10	0.17	0%	0.59	F
	PCR-RFLP	1.15	0.82-1.61	0.42	67%	0.005	R
AA vs. GA + GG							
Overall		1.09	0.80-1.49	0.58	76%	<0.0001	R
Ethnicity	Caucasian	0.72	0.51-1.03	0.07	0%	0.45	F
	Asian	1.22	0.86-1.73	0.28	76%	0.0003	R
Source of controls	PB	1.26	0.81-1.96	0.31	63%	0.04	R
	HB	1.05	0.62-1.79	0.85	83%	0.0006	R
Genotyping method	PCR-SSCP	1.09	0.60-1.98	0.77	69%	0.07	R
	PCR-RFLP	1.09	0.74-1.60	0.68	80%	0.0001	R
AA vs. GG							
Overall		1.09	0.73-1.63	0.66	75%	<0.0001	R
Ethnicity	Caucasian	0.97	0.63-1.48	0.87	0%	0.73	F
	Asian	1.12	0.67-1.87	0.66	81%	<0.0001	R
Source of controls	**PB**	**1.39**	**1.01-1.93**	**0.05**	**50%**	**0.11**	**F**
	HB	1.14	0.54-2.44	0.73	85%	0.0001	R
Genotyping method	PCR-SSCP	0.84	0.58-1.23	0.37	47%	0.17	F
	PCR-RFLP	1.14	0.70-1.87	0.60	78%	0.0001	R
GA vs. GG							
Overall		1.03	0.80-1.32	0.81	52%	0.04	R
Ethnicity	**Caucasian**	**1.49**	**1.06-2.10**	**0.02**	**0%**	**0.65**	**F**
	Asian	0.92	0.70-1.21	0.56	45%	0.09	R
Source of controls	PB	1.02	0.76-1.36	0.90	0%	0.44	F
	HB	1.16	0.72-1.87	0.54	72%	0.01	R
Genotyping method	PCR-SSCP	0.79	0.57-1.10	0.16	0%	0.97	F
	PCR-RFLP	1.12	0.83-1.50	0.45	53%	0.05	R

F-fixed effects model; R-random effects model.

**Table 4:** Association between cyclin D1 (CCND1) G870A polymorphism and type, Sex and H. pylori infection of the gastric cancer patients based on dominant models

Subgroup analyses	AA + GA vs. GG						
	Heterogeneity						
	OR	95%CI	P value	I^2^	P value	Effects model	No. of studies
Type							
cardiac	0.9	0.60-1.36	0.63	0%	0.88	F	2
non-cardiac	1.33	0.49-3.59	0.58	88%	0.0002	R	3
Sex							
**Male**	**0.5**	**0.33-0.76**	**0.001**	**0%**	**0.75**	**F**	**2**
Female	0.79	0.28-2.23	0.66	71%	0.07	R	2
H. pylori infection							
Positive	1.15	0.16-8.09	0.89	92%	0.0005	R	2
Negative	1.16	0.53-2.56	0.71	57%	0.13	F	2

### Sensitivity analyses

As shown in Table 1, all the studies conformed to the balance of HWE in controls except Kuo's (P<0.05), however, after performing the sensitivity analyses, When removing any of the articles, the overall outcomes were no statistically significant change, suggesting that this meta-analysis has good stability and reliability.

### Detection for heterogeneity

Heterogeneity among studies was obtained by Q statistic in the following genetic models: allele model (P<0.0001, I^2^ = 77%), the dominant model (P = 0.003, I^2^ = 66%), the recessive model (P<0.0001, I^2^ = 76%), the homozygous genetic model (P<0.0001, I^2^ = 75%), and the heterozygous genetic model (P = 0.04, I^2^ = 52%), the random-effects model was applied in these studies.

### Publication bias

**Figure 5:**
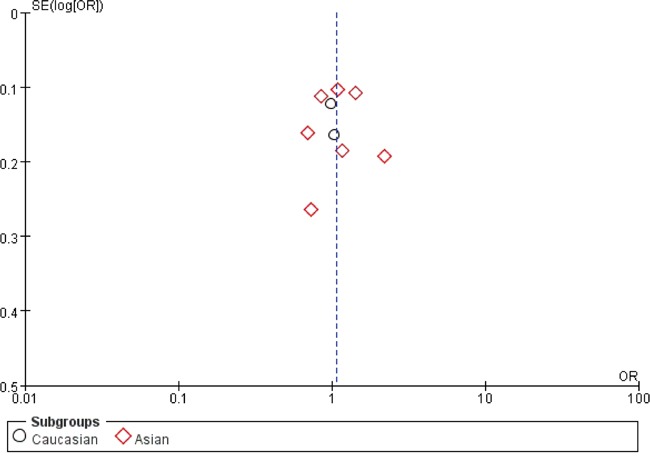
Funnel plot assessing evidence of publication bias from 9 studies (A vs. G) Abbreviations: SE, standard error; OR, odds ratio; A vs. G, Allele model.

We use Begg's funnel plot and Egger test to evaluate the published bias. As shown in Figure 5, the funnel plot is symmetrical, indicating that there is no significant publication bias in the total population. In our meta-analysis, no significant publication bias was found in the Begg's test and Egger's test (P>0.05).

## DISCUSSION

CCND1 alterations was reported to be frequently seen in GC and associated with its poor differentiation [22, 23]. The CCND1 polymorphism is a much concerned Single Nucleotide Polymorphism (SNP), for the G870A allele creates a variant splice transcript popular as “transcript b” by regulating mRNA [24–27]. Transcript b is constitutively nuclear in localization and may be more oncogene [28–30]. Previous functional studies have reported the relationship between cyclin D1 G870A polymorphism and the occurrence of GC, However, the conclusions are still inconclusive [21, 31]. Therefore, we carried out the meta-analysis on the whole included case-control researches to make a more accurate assessment of the relationship.

In our study, 9 studies were eventually included in our study, including 1813 cases and 2173 controls. [12–20]. In the total population, the pooled results indicated that there was not any relationships between G870A polymorphism and the occurrence of GC in any genetic model: Allele model (OR=1.07, 95% CI=0.88-1.30, P=0.51), dominant model (OR=1.07, 95% CI=0.81-1.41, P=0.65), recessive model (OR=1.09, 95% CI=0.80-1.49, P=0.58), homozygous genetic model (OR=1.09, 95% CI=0.73-1.63, P=0.66), heterozygote comparison (OR=1.03, 95% CI=0.80-1.32, P=0.81).

The subgroup study stratified by ethnicity showed an increased GC risk in Caucasian based on heterozygote comparison. while, there was not any genetic models attained statistical correlation in Asians (Table 3). We found an increased GC risk in population based (PB) stratified analyses by Source of controls (Figure 3). However, no statistically significant association in hospital-based (HB) (Table 3). When stratifying by the type, Sex and H. pylori infection in dominant model, Interestingly, we found the opposite result in Male (Figure 4). While, not any relationships between CCND1 G870A polymorphism and GC risk in any other subgroups (Table 4).

In a previous meta-analysis by Chen et al [21], they found the cyclin D1 G870A allele can significantly promote the risk of GC in Caucasian based on heterozygote comparison which consistent with our findings. They also find the same risk in Males which was contrary to our findings. They also found the cyclin D1 G870A allele can significantly promote the risk of GC for population with H. pylori infection, which was not shown in our studies. It should be pointed out that our results are different from Chen's analysis. The contradiction may be due to the difference in the sample size and the differences in race. Only four papers were included in Chen's meta-analysis, while nine studies in our analysis.

Our meta-analysis has some limitations in the following aspects. First, Our study is a summary of the data. We did not verify it from the level of basic experiments. Second, We just included the published studies in our study. There may still be some published studies in line with the conditions. Third, the Selected papers were mostly from Asian population. Only two papers are about Caucasian population. Finally, just dominant model was used when stratifying by the type, Sex and H. pylori infection for the limitation of data. Data from a large sample of multiple centers based on Caucasian or African is still needed to confirm the relationship between cyclin D1 G870A polymorphism and GC risk.

In conclusion, our study suggests that CCND1 G870A polymorphism could increases the risk of GC in Caucasians and in general populations. While, CCND1 G870A polymorphism plays a possible protective role in GC among males. Data from a large sample of multiple centers is still needed to confirm our findings.

## MATERIALS AND METHODS

### Literature searching strategy

We searched PubMed, EMBASE, Web of science, the Cochrane Library for relevant studies published before June 12, 2015. The following keywords were used: CCND1/cyclin D1, variant/genotype/polymorphism/SNP, Gastric/stomach/cardia, cancer/carcinom*/neoplasm*/tumor and the combined phrases for all genetic studies on the association between the cyclin D1 G870A polymorphism and GC risk. The reference lists of all articles were also manually screened for potential studies. Abstracts and citations were screened independently by two authors, all the agreed articles need a second screen for full-text reports. The searching was done without restriction on language.

### Selection and exclusion criteria

Inclusion criteria: A study was included in this meta-analysis if it meet the following criteria: *i*) independent case-control studies for humans; *ii*) the study evaluated the association between cyclin D1 polymorphism and gast*ric cancer risk; iii) has a*vailable genotype frequencies in cancer cases and control subjects for risk estimate. We excluded comments, editorials, systematic reviews or studies lacking sufficient data. If the publications were duplicated or shared in more than one study, the most recent publications were included. All identified studies were screened by two investigators independently. What's more, there were no limitation for publication language.

### Data extraction and synthesis

We used endnote bibliographic software to construct an electronic library of citations identified in the literature search. All the PubMed, EMBASE, Web of science and the Cochrane Library searches were performed using Endnote; duplicates were found automatically by endnote and deleted manually. All data extraction were checked and calculated twice according to the inclusion criteria listed above by two independent investigators. Data extracted from the included studies were as follows: First author, year of publication, country, ethnicity, Study design, Source of controls, Genotyping method and evidence of HWE in controls. A third reviewer would participate if some disagreements were emerged, and a final decision was made by the majority of the votes.

### Statistical analysis

All statistical analyses were performed using STATA version 11.0 software (StataCorp LP, College Station, TX) and Review Manage version 5.2.0 (The Cochrane Collaboration, 2012). Hardy-Weinberg equilibrium (HWE) was assessed by χ^2^ test in the control group of each study [32]. The strength of associations between the cyclin D1 polymorphism and GC risk were measured by odds ratio (ORs) with 95% confidence interval (CIs). Z test was used the to assess the significance of the ORs, I^2^ and Q statistics was used to determine the statistical heterogeneity among studies. A random-effect model was used if P value of heterogeneity tests was no more than 0.1 (P ≤ 0.1), otherwise, a fixed-effect model was selected [32, 33]. Sensitivity analyses were performed to assess the stability of the results. We used Begg's funnel plot and Egger's test to evaluate the publication bias [34, 35]. The strength of the association was estimated in the allele model (A vs. G), the dominant model (AA + GA vs. GG), the recessive model (AA vs. GA + GG), the homozygous genetic model (AA vs. GG), and the heterozygous genetic model (GA vs. GG), respectively. P < 0.05 was considered statistically significant. We performed subgroup according to Ethnicity, Source of controls, Genotyping method, type of cancer, gender and H. pylori infection.
